# Inhalative preconditioning with hydrogen sulfide attenuated apoptosis after retinal ischemia/reperfusion injury

**Published:** 2011-05-07

**Authors:** Julia Biermann, Wolf A. Lagrèze, Nils Schallner, Christian I. Schwer, Ulrich Goebel

**Affiliations:** 1 University Eye Hospital Freiburg, Freiburg, Germany; 2 Department of Anaesthesiology and Critical Care Medicine, University Medical Center, Freiburg, Germany

## Abstract

**Purpose:**

Retinal ischemia/reperfusion (I/R) injury plays an important role in the pathophysiology of various ocular diseases. Retinal ganglion cells (RGCs) are particularly vulnerable to ischemia. Hydrogen sulfide (H_2_S) was recently shown to be neuroprotective in the brain and retina due to its antiapoptotic effects. Rapid preconditioning of retinal neurons by inhaled H_2_S before I/R injury may reduce apoptosis in the rat retina.

**Methods:**

I/R injury was created on the left eye of rats (n=8) with or without inhaled H_2_S preconditioning (80 ppm) for one hour before ischemia. Densities of fluorogold prelabeled RGCs were analyzed 7 days after injury in retinal whole mounts. Retinal tissue was harvested to analyze protein expression of heat shock protein (HSP)-90 and the mitogen-activated protein kinases (MAPKs) c-jun N-terminal kinase (JNK), extracellular signal-regulated kinase (ERK)1/2 and p38 to elucidate a possible pathway of neuroprotection. DNA binding activity of the transcription factors nuclear factor-kappa-light-chain-enhancer of activated B-cells (NF-κB), cyclic adenosine monophosphate response element binding protein (CREB), and heat shock element (HSE), as well as caspase-3 cleavage and activity, were determined. Retinal sections were further assessed using anti–glial fibrillary acidic protein staining.

**Results:**

RGC death after I/R injury decreased by 41.5% after H_2_S preconditioning compared to room air (p<0.001). H_2_S inhalation before ischemia reduced caspase-3 cleavage (p<0.001) and attenuated caspase-3 activity (p<0.001). Furthermore, HSP-90 expression was significantly elevated in the retina after H_2_S preconditioning. NF-κB but not CREB or HSE showed specific, H2S-dependent regulation, as well as the MAPKs ERK1/2 and JNK but not p38.

**Conclusions:**

H_2_S preconditioning mediates antiapoptotic effects in retinal I/R injury, thus exhibiting neuroprotection. Based on these observations, H_2_S could represent a novel and promising therapeutic agent to counteract neuronal injuries in the eye. Further studies are needed to prove H_2_S’s neuroprotective propensity using a postconditioning approach.

## Introduction

Ischemia/reperfusion (I/R) injury plays an important role in the pathophysiology of eye diseases such as diabetic retinopathy [1], retinal vascular occlusion [2], anterior optic neuropathy [3], and possibly glaucoma [4,5]. It may ultimately lead to neuronal death by inducing apoptosis [6] or necrosis [7]. Of the different retinal neurons, retinal ganglion cells (RGCs) are particularly vulnerable to ischemia [8,9]. An option to increase the organ’s resistance to such injury is the concept of ischemic preconditioning (IPC), first introduced by Murry et al. [10]. The concept implies that a brief subcritical ischemic or chemical challenge could mobilize intrinsic protective mechanisms, increasing tolerance to subsequent critical ischemia. This induction of ischemic tolerance has gained attention as a robust neuroprotective mechanism. Two types of preconditioning have been identified to develop the tolerant state, one that occurs very rapidly (within 1 h) [11,12] and a second that develops slowly (over days) [13]. In the retina, the delayed type of IPC (brief episodes of ischemia or systemic hypoxia) has been well established [6,14]. We recently detected a neuroprotective effect using rapid IPC with inhalative carbon monoxide (CO) before ischemia in the rat retina [15].

Hydrogen sulfide (H_2_S) is generally thought to be a poisonous gas [16]. However, relatively high endogenous levels of H_2_S have recently been measured in the brains of rats and humans [17]. As H_2_S is produced in the brain, it may play a role in synaptic transmission. Intensive research over the last several years revealed its involvement in neurotransmission, neuroprotection, and smooth muscle relaxation [18]. After Blackstone et al. [19,20] demonstrated prolonged survival of mice under hypoxic conditions after H_2_S exposure, a series of investigations was launched to analyze the cyto- and organ-protective effects of H_2_S in detail; however, these are not yet fully understood. The physiologic effects of endogenous H_2_S range from Ca^2+^- and calmodulin-mediated neuronal excitation [21] to vasorelaxation via stimulation of ATP (ATP)-sensitive potassium channels [22]. Another effect on neurons is that H_2_S protects them by increasing levels of the major and potent antioxidant glutathione through the enhancement of γ-glutamylcysteine activity [23]. Especially in myocardial I/R models, the protective effects of H_2_S have been proven in combination with the beneficial function of myocardiac muscle cells via their antiapoptotic and anti-inflammatory properties [24-27]. In vitro, the neuroprotective properties of H_2_S seem to partly involve the mitochondrial function, as well as the heat shock response and a modulation of the mitogen-activated protein kinases (MAPKs) [23,28,29]. Thus, it appears that the effects of H_2_S through these intracellular signaling pathways are orientated toward neuroprotection against immunological (e.g., inflammation) and oxidative (e.g., reactive oxygen species) stress and the promotion of survival and development [30].

Only recently, Osborne et al. [31] reported a neuroprotective effect of the H_2_S donor ACS67, a derivative of latanoprost acid, after retinal ischemia and after an oxidative insult to RGC-5 cells in culture. To date, there have been no data concerning possible protective effects of inhaled H_2_S in relation to retinal cells. We chose the rapid form of preconditioning to investigate H_2_S-related mechanisms in apoptosis and neuronal survival in the rat retina after I/R injury. As no specific receptor or pathway has been identified as mediating H_2_S’s cytoprotective effects so far, we screened some of the common pathways and molecules with regard to neuroprotection. Besides the analysis of the effector caspase-3 and the transcription factors cyclic adenosine monophosphate (cAMP) response element binding protein (CREB) and nuclear factor-kappa-light-chain-enhancer of activated B-cells (NF-κB), we studied the modulation of the phosphorylated MAPK c-Jun N-terminal kinase (JNK), extracellular signal-regulated kinase 1/2 (ERK1/2) and p38 kinase, as well as the heat shock response. We hypothesized that inhalation of H_2_S before I/R injury may reduce apoptosis, thus exerting neuroprotective effects. We conducted this study in an identical way in the exact same animal model using an identical stressful stimulus as described for CO [15] to make a comparison of the obtained results possible, and to examine whether these gaseous neurotransmitters exert their effects through different molecular pathways.

## Methods

### Animals

Adult male and female Sprague-Dawley rats (300–350 g bodyweight; Charles River, Sulzfeld, Germany) were used in this study. Animals were fed a standard rodent diet ad libitum while kept on a 12h:12h light-dark cycle. All procedures involving the animals concurred with the ARVO Statement for the Use of Animals in Ophthalmic and Vision Research and were approved by the Committee of Animal Care of the University of Freiburg. All types of surgery and manipulations were performed under general anesthesia with isoflurane/O_2_ for retrograde labeling with fluorogold (FG) or with a mixture of intraperitoneally administered ketamine 50 mg/kg (Ceva-Sanofi, Duesseldorf, Germany) and xylazine 2 mg/kg (Ceva-Sanofi) for the ischemia and reperfusion experiment. Body temperature was maintained at 37 °C±0.5 °C with a heating pad and a rectal thermometer probe. After surgery, buprenorphine (Temgesic^®^ 0.5 mg/kg; Essex Pharma, Solingen, Germany) was applied intraperitoneally to prevent pain. During recovery from anesthesia, the animals were placed in separate cages, and gentamicin ointment (Refobacin; Merck, Darmstadt, Germany) was applied on ocular surfaces and skin wounds. Eight animals per group were used for RGC quantification and molecular analysis. For proteomics and electrophoretic mobility shift assay (EMSA), the tissue was harvested 24 h after I/R injury.

### Retrograde retinal ganglion cell labeling

Deeply anesthetized rats were placed in a stereotactic apparatus (Stoelting, Kiel, Germany), and the skin overlying the skull was cut open and retracted. The lambda and bregma sutures served as landmarks for drilling six holes. FG (7.8 µl; Fluorochrome LLC; Denver, CO) dissolved in dimethyl sulfoxide (DMSO) was injected into both superior colliculi. To ensure proper RGC labeling, animals were allowed 7 days for retrograde transport of FG before further experimental intervention.

### Hydrogen sulfide (H_2_S) preconditioning and retinal ischemia/reperfusion injury

To evaluate the neuroprotective effect of inhalative H_2_S, animals were randomly assigned to receive preconditioning with room air or room air supplemented with 80 ppm H_2_S (Air Liquide, Kornwestheim, Germany), both for 1 h in a sealed chamber before the experiment. During this inhalation, rats were awake and freely moving in their cages. They were anesthetized intraperitoneally (the procedure lasted approximately 10 min) immediately after preconditioning, and the anterior chamber of the left eye was cannulated with a 30-gauge needle connected to a reservoir containing 0.9% NaCl. Intraocular pressure was increased to 120 mmHg for 60 min, and ocular ischemia was confirmed by interruption of the ocular circulation, as described previously [32]. Thereafter, the cannula was immediately retracted, and the adequacy of retinal reperfusion was confirmed visually by an ophthalmoscope. The right eyes served as controls. Rats that did not recover from retinal perfusion 3 min after the end of the ischemic period and those with lens injury (which prevents RGC death and promotes axonal regeneration [33]) were excluded from the investigation.

### Retinal ganglion cell quantification

Animals were sacrificed by CO_2_ inhalation 7 days after ischemia. Retinal tissue was immediately harvested and further processed for whole-mount preparation in ice-cold Hanks balanced salt solution. Retinas were carefully placed on a nitrocellulose membrane with the ganglion cell layer (GCL) on top. After the vitreous body was removed, retinas were fixed in 4% paraformaldehyde for 1 h and then embedded in mounting media (Vectashield; AXXORA, Loerrach, Germany). The densities of FG-positive RGCs were determined in a blinded fashion with a fluorescence microscope (AxioImager; Carl Zeiss, Jena, Germany) and the appropriate bandpass emission filter (FG: excitation/emission, 331/418 nm), as previously described [34]. Briefly, we photographed three standard rectangular areas (each measuring 0.200 mm×0.200 mm=0.04 mm^2^) at 1, 2, and 3 mm from the optic disc in the central region of each retinal quadrant. Hence, we evaluated an area measuring 0.48 mm^2^/retina (12×0.04 mm^2^), which represents approximately 1% of the rat retina, assuming an average area per retina of approximately 50 mm^2^ in rats. To determine the number of cells per square millimeter, we multiplied the number of analyzed cells/0.04 mm^2^ by 25. Secondary FG-stained activated microglial cells were separated by morphological criteria after RGC phagocytosis and were excluded from examination. All averaged data are presented as mean RGC density (cells/mm^2^) ±standard deviation (SD).

### Western blot analysis

Total retinal cell lysates were prepared 24 h after ischemia by the addition of 100 µl sodium dodecyl sulfate (SDS) buffer (250 mM Tris [pH 6.8], 10% SDS, 500 mM dithiothreitol, 50% glycerol, and 0.5% bromophenol blue). Five micrograms of total cellular extracts were separated on a 7.5% SDS polyacrylamide gel. Proteins were transferred to a nitrocellulose membrane (Bio-Rad, Hercules, CA), and the membranes were blocked with 5% skim milk in Tween-20/phosphate-buffered saline and incubated with the indicated protein-specific antibodies (pro-caspase-3 #9662, cleaved caspase-3 #9661, p-JNK #9251, p-ERK #9101, and p-p38 #9211; Cell Signaling, Danvers, MA) overnight at 4 °C. After incubation with a horseradish peroxidase-conjugated antirabbit immunoglobulin antibody, proteins were visualized with an enhanced chemiluminescence kit (GE Healthcare, Little Chalfont, UK). For normalization, blots were reprobed with a housekeeping antibody or total form of MAPK (glyceraldehyde 3-phosphate dehydrogenase [GAPDH] #2118, JNK #9258, ERK #9102 and p38 #9228; Cell Signaling) and were analyzed by laser scanning densitometry (Personal Densitometer; GE Healthcare).

### Fluorogenic caspase-3 activity assay

Fluorogenic caspase activity assay was performed 24 h after ischemia using full retinal protein extracts [35]. Results are given in arbitrary fluorescent units (RFUs) ±SD.

### Enzyme-linked immunosorbent assay

Full retinal protein was extracted 24 h after ischemia, and enzyme-linked immunosorbent assay (ELISA) was performed according to the manufacturer’s instruction (heat shock protein [HSP]-90α, StressXPress SPA-835; Biomol, Hamburg, Germany). Protein concentration was determined using the Bradford assay (Bio-Rad Laboratories, Munich, Germany). Results are given in mean±SD.

### Electrophoretic mobility shift assay

EMSA was performed 24 h after ischemia with [γ-^32^P]-ATP-labeled NF-κB, CREB, and HSE oligonucleotides [35] using the NF-κB consensus sequence 5′-AGT TGA GGG GAC TTT CCC AGG-3′, the CREB consensus sequence 5′-AGA GAT TGC CTG ACG TCA GAG ACG TAG-3′, and the HSE consensus sequence 5′-CTA GAA GCT TCT AGA AGC TTC TAG-3′. Supershift analysis was performed using NF-κB p50 and p65 antibodies (Sc-114x and Sc-8008x; Santa Cruz Biotechnologies, Santa Cruz, CA) and AP-1 c-fos antibody (Sc-253x; Santa Cruz Biotechnologies). Sensitivity was achieved using the respective unlabeled oligonucleotide or AP-1 as a control. Results are given in relative densitometric units (mean±SD).

### Immunohistochemistry

Rat eyes (n=2) were enucleated 7 days after ischemia, embedded in compound (Tissue-Tek; Sakura-Finetek, Torrance, CA), and frozen in liquid nitrogen. Frozen sections (10 µm) were cut through the middle third of the eye and collected on gelatinized slides. Immunohistochemistry was performed according to standardized protocols with monoclonal antibodies against glial fibrillary acidic protein (GFAP; dilution 1:400; Sigma), which was then conjugated with the corresponding secondary antibody (Cy2™, dilution 1:200; Jackson ImmunoResearch, West Grove, PA). The nuclei of retinal cells were stained with 4’,6-diamino-2-phenylindole dihydrochloride hydrate (Sigma) added to the embedding medium (Mowiol; Calbiochem, San Diego, CA). Slides were examined under a fluorescence microscope (Axiophot; Carl Zeiss, Jena, Germany).

### Statistical analysis

Data were analyzed with a computerized statistical program (SigmaStat for Windows Version 3.1; Systat Software Inc., San Jose, CA). We intended to detect a 50% reduction in the H_2_S-mediated protective effects. Assuming an expected SD of 15% based on previously published data, an a priori power analysis (a=0.05 with the two-sided hypothesis, b=0.1, power 90%) indicated that a sample size of seven animals per group would be sufficient to detect such a reduction. The results are presented as means (±SD) after normal distribution of data had been verified. One-way ANOVA for repeated measurements was used for between-group comparisons with the post hoc Tukey–Kramer test. A p<0.05 was considered statistically significant. Autoradiographies of EMSA and western blot analysis were evaluated by volume quantification and the local median of protein expression, and normalization against background or loading control using two-dimensional scanning (Personal Densitometer; GE Healthcare, Freiburg, Germany).

## Results

All animals survived the experiments and were included in the data analysis. No sign of disease or harm was recognized in any of them. The untreated right eye in every animal served as a control for each experiment.

### H_2_S preconditioning attenuated retinal ganglion cell death after ischemia/reperfusion injury

**Figure 1:**
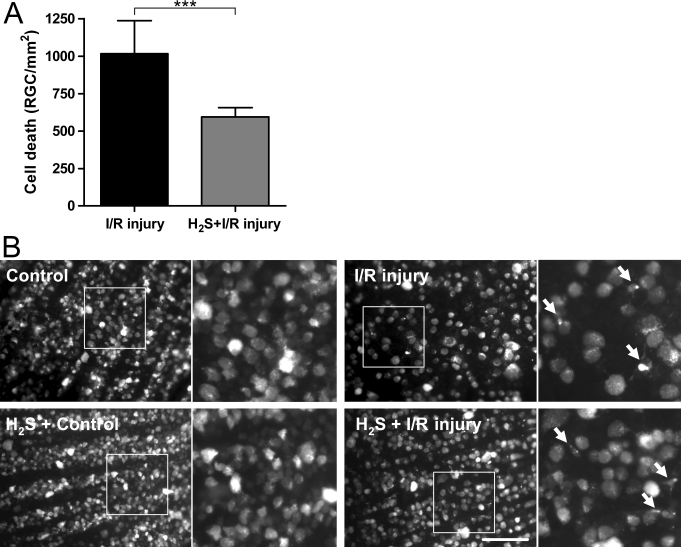
Attenuated retinal ganglion cell death after hydrogen sulfide preconditioning. **A**: Retinal ganglion cell (RGC) loss (RGC/mm^2^) presented as mean difference to individual control. Data are presented as mean±SD of eight experiments. Hydrogen sulfide (H_2_S) preconditioning decreased RGC death by 41.5% compared to room air seven days after ischemia/reperfusion (I/R) injury (***p<0.001). **B**: RGC densities were not significantly different in controls exposed to room air or H_2_S. Fluorogold (FG)-positive RGCs can be identified by morphological criteria (large round cell body, no processes, almost homogeneously labeled). In I/R treated eyes, many RGCs died and activated microglia cells (small cellular body, branching processes, inhomogeneously labeled; denoted with arrows in the extracts) stained FG-positive after RGC phagocytosis. H_2_S preconditioning partly antagonized this effect, leading to significantly higher cell densities. The scale bar represents 100 µm.

RGC densities were counted to analyze the effect of H_2_S in the context of ischemia and reperfusion. As in control retinas exposed to room air, all RGCs stained FG-positive in the H_2_S control group 7 days after I/R injury on the opposite eye (2,573±261 or 2,602±258 RGC/mm^2^, respectively), data not shown. I/R injury with or without H_2_S preconditioning reduced RGC densities to 2,008±181 and 1,557±207 RGC/mm^2^, respectively. Thus, I/R injury after room air inhalation led to a ~40% reduction of viable RGCs (1,016±221 dead RGC/mm^2^) compared to untreated control eyes (p<0.001; Figure 1A). Preconditioning with inhalative H_2_S attenuated RGC death after I/R injury (only ~23% reduction of viable RGCs, 594±178 dead RGC/mm^2^) compared to H_2_S-exposed controls (p<0.001; Figure 1A). In summary, 7 days after I/R injury, RGC death decreased by 41.5% due to H_2_S preconditioning compared to room air (p<0.001). In I/R treated eyes, many RGCs died and activated microglia cells (denoted with arrows) stained FG-positive after RGC phagocytosis (Figure 1B).

### H_2_S reduced apoptosis in retinal tissue after ischemia/reperfusion injury

**Figure 2:**
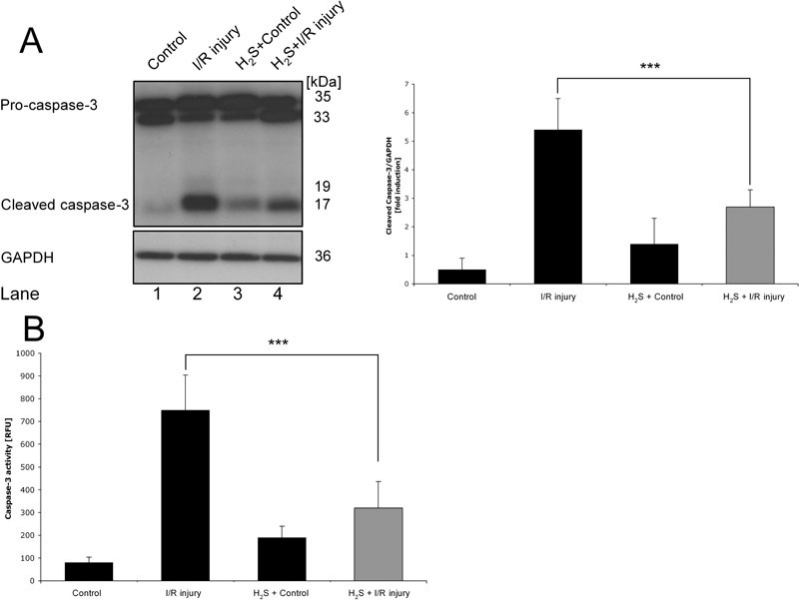
Hydrogen sulfide preconditioning–mediated antiapoptotic effects. **A**: Effect of hydrogen sulfide (H_2_S) preconditioning on caspase-3 activation 24 h after unilateral ischemia. Pro-caspase-3 and caspase-3 levels were determined using specific antibodies. Histograms represent the densitometric ratio of caspase-3 cleavage compared with loading control (glyceraldehyde 3-phosphate dehydrogenase [GAPDH]). The amount of pro-caspase-3 and protein loading seemed comparable in all groups (lanes 1–4). Compared to control, ischemia/reperfusion (I/R) injury led to a significant cleavage from pro-caspase-3 to active caspase-3 (lane 1 versus 2). H_2_S preconditioning before I/R injury significantly reduced cleavage of pro-caspase-3 to caspase 3 (lane 4 versus 2; ***p<0.001). Data are presented as mean±SD of five experiments. **B**: Fluorogenic caspase-3 assay (DEVDase assay) of full retinal protein lysates 24 h after I/R injury. Caspase-3 activity was low in control eyes (room air) and was not significantly affected by H_2_S inhalation in controls. I/R injury increased the activity (p<0.001 compared with control eye). In contrast, preconditioning with inhaled H_2_S significantly reduced caspase-3 activity in ischemic tissue. Results are given in RFUs. Data are presented as mean±SD of eight experiments. ***p<0.001 I/R injury versus H_2_S + I/R injury.

While investigating the effects of I/R injury and H_2_S on retinal apoptosis, we analyzed the cleavage of caspase-3 and the caspase-3 activity in the retina 24 h after ischemia. Compared to control, I/R injury led to a significant cleavage from pro-caspase-3 to caspase-3 (Figure 2A, lane 1 versus 2). H_2_S preconditioning in control animals had no significant effect (Figure 2A, lane 3). H_2_S preconditioning before I/R injury significantly reduced cleavage of pro-caspase-3 to caspase 3 (Figure 2A, lane 4 versus 2; p<0.001). In agreement with this data, caspase-3 activity was low in control eyes and was not affected by H_2_S inhalation in the control eyes of H_2_S-preconditioned animals (Figure 2B, 99±34 versus 180±73 RFU). I/R injury increased the activity to 750±154 RFU (p<0.001 compared with control eyes). In contrast, preconditioning with inhaled H_2_S reduced caspase-3 activity significantly (Figure 2B, 320±117 versus 750±154 RFU; p<0.001).

### H_2_S attenuated glial fibrillary acidic protein expression in the retina after ischemia/reperfusion injury

**Figure 3:**
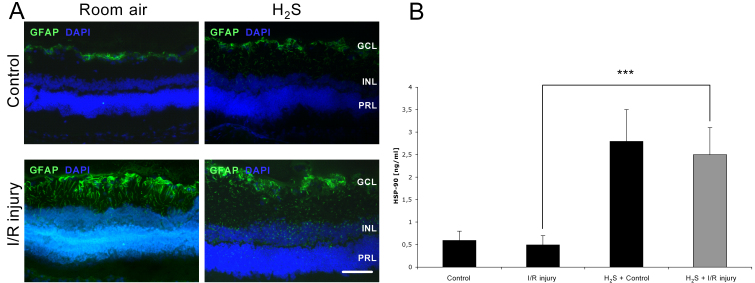
Hydrogen sulfide–attenuated glial fibrillary acidic protein expression and increased heat shock protein (HSP)-90 expression in retinal tissue. Effect of hydrogen sulfide (H_2_S) preconditioning on retinal glial fibrillary acidic protein (GFAP; **A**) and HSP-90 (**B**) expression. **A**: Cross-sections of the retinas 7 days after unilateral ischemia/reperfusion (I/R) injury. In controls with and without H_2_S preconditioning, GFAP was only positive in Müller cells and astrocytes in the ganglion cell layer (GCL). After ischemia, GFAP was upregulated in Müller cells. Their processes, extending through all retinal layers, became strongly GFAP positive. This upregulation seemed to be stronger in the I/R injury + room air group. The scale bar represents 50 µm. **B**: In nonpreconditioned eyes (control and ischemic), retinal HSP-90 expression remained at the baseline level. H_2_S preconditioning in control eyes significantly increased HSP-90 expression. H_2_S inhalation before I/R injury significantly induced retinal HSP-90 expression compared with I/R injury alone (***p<0.001). Data are presented as mean±SD of eight experiments.

Histological analysis of the retina was performed 7 days after unilateral I/R injury. In controls with and without H_2_S preconditioning, GFAP was only positive in Müller cells and astrocytes in the GCL (Figure 3A). After ischemia, GFAP was upregulated in Müller cells. Their processes, extending through all retinal layers, became strongly GFAP-positive. This upregulation seemed to be stronger in the I/R injury plus room air group, thus correlating with the degree of retinal damage.

### H_2_S increased HSP-90α expression in retinal tissue

In nonpreconditioned eyes (control and I/R injury), retinal HSP-90α expression remained at baseline levels (Figure 3B, 0.6±0.2 and 0.5±0.3 ng/ml, respectively) 24 h after unilateral ischemia. H_2_S preconditioning significantly increased HSP-90α expression in control (Figure 3B, 2.8±0.7; p<0.001 versus control) and ischemic eyes (Figure 3B, 2.5±0.6 versus 0.5±0.3 ng/ml, p<0.001); thus, this upregulation seems to be independent of I/R injury.

### H_2_S reduced DNA-binding activity of NF-κB after ischemia

**Figure 4:**
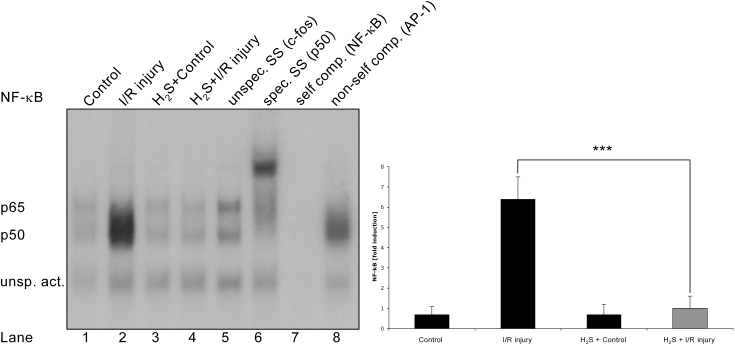
Hydrogen sulfide counteracted ischemia/reperfusion-induced DNA-binding activity of nuclear factor-kappaB . Effect of hydrogen sulfide (H_2_S) on the DNA-binding activity of nuclear factor-kappaB (NF-κB) in retinal tissue 24 h after unilateral ischemia. Control eyes with and without H_2_S preconditioning did not reveal any DNA binding of NF-κB (lanes 1, 3). Compared to control, ischemia/reperfusion (I/R) injury increased the DNA binding of NF-κB significantly (lane 1 versus 2; sixfold induction versus control, p<0.001). H_2_S inhalation before I/R injury counteracted the ischemia-induced DNA binding of NF-κB completely (lane 4 versus 2, 5.8-fold reduction versus I/R injury, ***p*<*0.001). Specific supershift analysis revealed that p50 is the main part of the transactive NF-κB domain (lane 6), while the unspecific antibody (c-fos [AP-1]) did not lead to a supershift. The oligo’s sensitivity was demonstrated by competition experiments with unlabeled NF-κB (lane 7) and unlabeled AP-1 (lane 8). The histogram represents the densitometric ratio of NF-κB compared with the control group. Data are presented as mean±SD of five experiments.

EMSA was performed 24 h after ischemia to analyze the binding activity of transcription factors. Control and I/R injured eyes with and without H_2_S preconditioning did not reveal any DNA binding of CREB or HSE (data not shown). Compared to control, I/R injury increased the DNA binding of NF-κB significantly (Figure 4, lane 1 versus 2; sixfold induction versus control, p<0.001). While H_2_S preconditioning without injury did not alter DNA binding, H_2_S inhalation before I/R injury counteracted the DNA binding of NF-κB completely (Figure 4, lane 4 versus 2, 5.8 fold reduction versus I/R injury, p<0.001). Specific supershift analysis revealed that p50 is the main part of the transactive NF-κB domain (Figure 4, lane 6), while the unspecific antibody (c-fos [AP-1]) did not lead to a supershift. The oligo’s sensitivity was demonstrated by competition experiments with unlabeled NF-κB (Figure 4, lane 7) and unlabeled AP-1 (Figure 4, lane 8).

### H_2_S mediated differential mitogen activated protein kinase regulation in the retina

**Figure 5:**
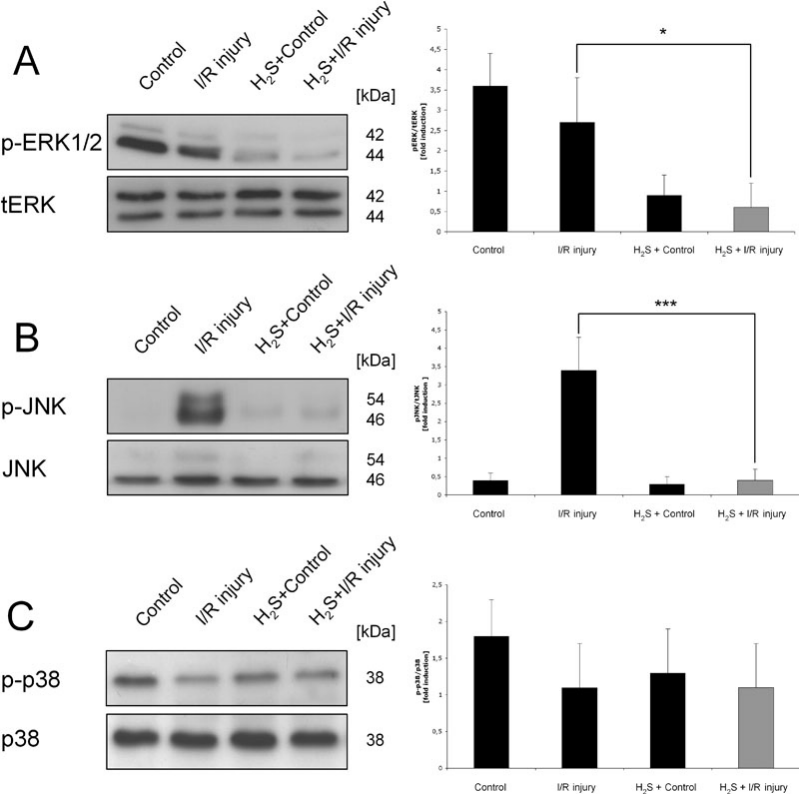
Hydrogen sulfide–induced differential mitogen-activated protein kinase regulation in the retina. Effect of hydrogen sulfide (H_2_S) preconditioning on mitogen-activated protein kinases (MAPKs) phosphorylated (p)- extracellular signal-regulated kinase (ERK)1/2 (**A**), p- c-jun N-terminal kinase (JNK; **B**), and p-p38 (**C**) 24 h after unilateral ischemia. MAPK levels were determined using specific antibodies. The histograms represent the densitometric ratio of MAPKs compared with its nonphosphorylated form, total (t)ERK1/2, JNK, or p38. **A**: p-ERK1/2 is suppressed through inhalational H_2_S preconditioning. While p-ERK was baseline activated in the nonpretreated animals (control and ischemia/reperfusion [I/R] injury), H_2_S significantly inhibited ERK1/2 phosphorylation in both control and ischemic retinas (fourfold reduction in H_2_S + I/R injury versus I/R injury; *p<0.05). **B**: I/R injury per se significantly increased the phosphorylation of the JNK (3.8-fold induction in I/R injury versus control; p<0.001). Again, preconditioning with inhalative H_2_S inhibited JNK phosphorylation completely (3.7-fold reduction in H_2_S + I/R injury versus I/R injury; ***p<0.001). **C**: The phosphorylation of p38 MAPK was comparable in all groups without significant differences. Data are presented as mean±SD of eight experiments.

The MAPK ERK1/2 is suppressed through inhalational H_2_S preconditioning. While p-ERK was baseline activated in the nonpretreated animals (control and I/R injury), H_2_S significantly inhibited ERK1/2 phosphorylation in both control and ischemic retinas (Figure 5A, fourfold reduction in H_2_S + I/R injury versus I/R injury; p<0.05). In contrast, I/R injury per se significantly increased the phosphorylation of JNK (Figure 5B, 3.8 fold induction in I/R injury versus control; p<0.001). Again, preconditioning with inhalative H_2_S inhibited JNK phosphorylation completely (Figure 5B, 3.7 fold reduction in H_2_S+I/R injury versus I/R injury; p<0.001). The phosphorylation of p38 MAPK was comparable in all groups without significant differences (Figure 5C).

## Discussion

The main findings of this experimental in vivo study can be summarized as follows: Ischemia and reperfusion cause significant neuronal injury in the retina, characterized by a reduced number of viable RGCs; inhalation of H_2_S before ischemia reduces RGC loss and attenuates caspase-3 cleavage as well as caspase-3 activity, thus acting antiapoptotically; H_2_S preconditioning further reduces GFAP activation in the retina and increases the protein expression of HSP-90α; possible mechanisms of the H_2_S-mediated protection are suppression of the transcription factor NF-κB and differential expression of MAPKs. These findings support our hypothesis that rapid preconditioning with inhaled H_2_S mediates antiapoptotic and thus neuroprotective effects in the rat retina after I/R injury.

The molecular mechanisms of rapid preconditioning are not fully understood; however, posttranslational modifications seem to play a key role in mediating neuroprotection in that form. In many models of delayed preconditioning, new protein synthesis is required, suggesting that subsequent changes in gene expression may underlie this form of preconditioning. However, it is clear that some changes in gene expression occur extremely rapidly, so there may be considerable overlap in the mechanisms of rapid and delayed preconditioning [36]. Rapid preconditioning has been previously observed in the central nervous system (CNS) [12,37], and was recently demonstrated by our laboratory in the rat retina for CO [15]. In some cases, neuroprotection after rapid IPC was not as robust or long lasting as that after delayed IPC [12]. To date, there are no data concerning possible protective effects of inhaled H_2_S preconditioning on retinal cells. In the present study, unilateral I/R injury resulted in a loss of ~40% of viable RGCs in the retina compared to uninjured controls. H_2_S preconditioning for 1 h before I/R injury reduced RGC loss by 41.5% on day 7 after ischemia compared with the I/R injury + room air group (Figure 1). This is a significant delay of cell death one week after I/R injury. However, the long-term effect of H_2_S preconditioning on RGC survival was not determined in this study. To suggest general neuroprotection in the retina, future studies should also address the survival promoting effect of H_2_S preconditioning on other retinal cells.

Previous studies in rat in vivo models—mainly in myocardial ischemia and reperfusion settings—have shown that H_2_S is able to modulate and prevent cell death [24,25]. Moreover, Tay et al. reported that H_2_S—as a chemical substrate derived from NaHS as a donor—protected neuronal cells from hypoxic damage by a K_ATP_/PKC/ERK and HSP-90 pathway [29]. Just recently, Osborne et al. first reported a neuroprotective action of the H_2_S donor ACS67 when administered intravitreally after retinal ischemia [31]. The present work revealed that inhaled H_2_S before I/R injury triggers neuronal protection by an antiapoptotic response. Caspase-3 is the effector caspase, connecting the cellular and mitochondrial apoptotic pathway. As described by Osipov et al., H_2_S is able to reduce caspase-3 cleavage, and subsequently caspase activity in a model of myocardial I/R injury [38]. Moreover, Osborne et al. found a reduction of apoptosis after an oxidative insult to RGC-5 cells in culture, following the activation of ACS67 [31]. In the same study, intravitreal ACS67 administration directly after ischemia counteracted retinal I/R injury, although the upregulation of caspase-3 and −8 mRNA levels were not diminished by ACS67 [31]. Our data revealed that H_2_S is able to attenuate caspase-3 cleavage and caspase-3 activity (Figure 2A,B). In accordance with this, the activation of Müller cells was reduced in retinal sections after H_2_S + I/R injury (Figure 3A).

NF-κB is a transcription factor known to mediate the stimulus-dependent induction of genes critical to the inflammation and survival of neurons. Activation of NF-κB appears to play an important role in retinal degeneration following retinal ischemia and reperfusion injury [39,40]. It has been demonstrated frequently that the inhibition of NF-κB activation protects retinal neurons in various animal models, e.g., ischemia [40], retinal degeneration [41], and glaucoma [42]. In the present work, I/R injury significantly induced the DNA binding of NF-κB, whereas H_2_S preconditioning before ischemia resulted in the abolished DNA binding of NF-κB, as shown via EMSA (Figure 4). Although there are fewer data concerning H_2_S involvement in the activation or suppression of NF-κB [43,44], our data strongly suggest that the H_2_S-mediated inhibition of NF-κB activation is one component of its neuroprotective action after retinal ischemia. It has been further demonstrated that the inhibition of MAPK may alter NF-κB activation, thus exerting neuroprotective effects [45,46].

CREB is a transcription factor known to mediate the stimulus-dependent expression of genes critical to neuronal survival. Recent studies indicate that CREB may be a key element in the acquisition of ischemic tolerance in the brain [47], and that IPC induces CREB activation and Bcl-2 expression in a neonatal ischemia model [48]. After retinal injury, CREB decreased in RGCs [49]. In our previous experiments using CO inhalation for IPC before retinal ischemia, we detected a more than threefold increase of CREB compared to controls [15]. In contrast, we were unable to detect increased DNA-binding activity of CREB in the present experiments. This is a clear hint that H_2_S mediates its protective properties at least in part through a different pathway than CO.

We have chosen the MAPKs p38, pERK1/2, and pJNK as targets worth analyzing, because the MAPKs are important in every cellular regulation, especially cellular protection. Furthermore, the MAPKs seem to play a crucial role in neuronal apoptosis; however, the data on the H_2_S-mediated phosphorylation of MAPKs are discordant. This study revealed that the phosphorylation and activation of the MAPKs JNK and ERK1/2 but not p38 show differential expression patterns after H_2_S preconditioning (Figure 5A-C). In the context of neuroprotection, the role of p38 regulation is controversial. An H_2_S-mediated antineuroinflammatory effect due to p38 inhibition was previously reported by Hu et al. [50]. In contrast, Dreixler et al. [51] reported a necessity for p38 activation in the context of retinal ischemia, and previous studies conducted by our laboratory demonstrated a phosphorylation of p38 by CO preconditioning before I/R injury [15]. Currently, there are fewer data about the effect of H_2_S on ERK1/2 in brain cells. Tay et al. [29] investigated H_2_S-related effects on hypoxia in neurons and concluded that ERK1/2 is needed for neuroprotection. In contrast, we found an H_2_S-induced inhibition of p-ERK1/2 in the ischemic retina. In other types of cells, H_2_S was found to stimulate ERK1/2 to induce apoptosis in human aorta smooth muscle cells [52], cardioprotection in the rat heart [53], and synthesis of proinflammatory cytokines in human monocytes [44]. In our model of retinal ischemia, H_2_S-mediated neuroprotection was associated with strongly diminished p-JNK activation. Likewise, Shi et al. found a rapid, time-dependent phosphorylation of JNK in cardial I/R injury and reported its significant inhibition after treatment with NaHS, an H_2_S donor [54]. Certainly, more experiments need to be done to clarify this pathway. In summary, the MAPKs are also regulated in a different way using H_2_S preconditioning compared to CO preconditioning in the same injury model. While p38 is not involved in H_2_S-mediated protection, JNK and ERK are expressed differentially.

HSPs are chaperone molecules that confer protein stability and help to restore protein native folding following heat shock and other stress. The most abundant HSP, HSP-90, is also involved in regulating the stability and function of several cell-signaling molecules. Following previous reports, HSPs are crucial for neuronal and nonneuronal survival, especially in ischemia [29,55] and in the mechanism of preconditioning [56]. It has been described that H_2_S contributes cellular protection via an induction of the heat shock response. In a model of neuronal hypoxia, HSP-90 was induced via H_2_S, revealing protective properties [29]. Our data confirmed this effect, showing a significant induction of the cytoprotective chaperone molecule HSP-90 by the application of H_2_S alone and in the context of I/R injury (Figure 3B). Interestingly, we were unable to detect increased DNA-binding activity of HSE in the present experiments, although the HSE and corresponding heat shock factor–1 are necessary elements involved in the heat shock response. However, the posttranslational protein modification represents another option to increase HSP-90 expression; this was not further investigated in the present study.

Despite various molecular mechanisms that have been described for H_2_S so far, no specific receptor has been identified. Current evidence suggests that under certain physiologic conditions and after neuronal injury, H_2_S plays a role in promoting cell survival by its neuroprotective effects on both neurons and glia in the CNS [30]. Current targets for H_2_S in the CNS include N-Methyl-D-aspartate (NMDA) receptors, as well as ATP-sensitive potassium and calcium channels, thereby promoting changes in neuronal and glial signaling, oxidative stress, and vascular regulation [18,57]. In contrast, H_2_S appears to cause or contribute to tissue damage [58], depending on concentration, cell type, and tissue specificity. The potential pathophysiological effects of H_2_S on other retinal cells such as photoreceptors need to be considered when using H_2_S. In the present study, we used room air supplemented with 80 ppm H_2_S for preconditioning, as in previous studies [20]. This dose was well tolerated by the rats.

Regarding the data presented here, it is tempting to propose a pathway for H_2_S-mediated antiapoptotic effects in the rat retina: The exogenous, inhalative application of H_2_S inhibits caspase-3 cleavage and caspase-3 activity. Our histological findings strengthen these results, giving morphological evidence of RGC survival, but also of reduced glial activation. Moreover, HSP-90 expression is increased due to H_2_S exposure. In part, these effects may be mediated by the inhibition of NF-κB activation and by a differential phosphorylation of the MAPKs ERK1/2 and JNK but not p38. Thus, H_2_S seems to mediate its effects via partly different pathways in comparison to CO. Depending on the gaseous molecule used, the mechanisms of protective organic preconditioning are different in molecular targets, mechanisms, and sites of action.
